# Effects of β-Si_3_N_4_ Seeds on Microstructure and Performance of Si_3_N_4_ Ceramics in Semiconductor Package

**DOI:** 10.3390/ma16124461

**Published:** 2023-06-19

**Authors:** Qiang Shen, Zhijie Lin, Junjie Deng, Hongxiang Chen, Xuan Chen, Jun Tian, Biliang Bao, Pinqiang Dai, Xudong Sun

**Affiliations:** 1 College of Materials Science and Engineering, Fujian University of Technology, Fuzhou 350118, China; shenq1117@163.com (Q.S.); dengjunjie31@163.com (J.D.); hungxchen@163.com (H.C.); tianj2003@126.com (J.T.); pqdai@126.com (P.D.); 2 Kazuo Inamori School of Engineering, New York State College of Ceramics, Alfred University, Alfred, NY 14802, USA; chenxuan1178238@gmail.com; 3 Fujian Minhang Electronics Co., Ltd., Nanping 353000, China; 18960668996@126.com; 4 College of Materials Science and Engineering, Foshan Graduate School of Northeastern University, Foshan 528311, China; xdsun@mail.neu.edu.cn

**Keywords:** ceramic substrate, fracture toughness, silicon nitride, β-Si_3_N_4_ crystal seed

## Abstract

Among the various ceramic substrate materials, Si_3_N_4_ ceramics have demonstrated high thermal conductivity, good thermal shock resistance, and excellent corrosion resistance. As a result, they are well-suited for semiconductor substrates in high-power and harsh conditions encountered in automobiles, high-speed rail, aerospace, and wind power. In this work, Si_3_N_4_ ceramics with various ratios of α-Si_3_N_4_ and β-Si_3_N_4_ in raw powder form were prepared by spark plasma sintering (SPS) at 1650 °C for 30 min under 30 MPa. When the content of β-Si_3_N_4_ was lower than 20%, with the increase in β-Si_3_N_4_ content, the ceramic grain size changed gradually from 1.5 μm to 1 μm and finally resulted in 2 μm mixed grains. However, As the content of β-Si_3_N_4_ seed crystal increased from 20% to 50%, with the increase in β-Si_3_N_4_ content, the ceramic grain size changed gradually from 1 μm and 2 μm to 1.5 μm. Therefore, when the content of β-Si_3_N_4_ in the raw powder is 20%, the sintered ceramics exhibited a double-peak structure distribution and the best overall performance with a density of 97.5%, fracture toughness of 12.1 MPa·m^1/2^, and a Vickers hardness of 14.5 GPa. The results of this study are expected to provide a new way of studying the fracture toughness of silicon nitride ceramic substrates.

## 1. Introduction

The development of third-generation semiconductors has induced a huge demand for ceramic substrates for power-integrated circuits [1,2,3,4]. Conventionally, Al_2_O_3_ has been utilized as the substrate in power-integrated circuits due to its cost-effectiveness. However, Al_2_O_3_ has a low toughness (3–5 MPa·m^1/2^) and thermal conductivity (18–24 W∙m^−1^∙K^−1^) and cannot effectively satisfy the requirements of high-power demand in energy and other emerging fields [5]. AlN is another ceramic substrate material that shows high thermal conductivity (150–270 W∙m^−1^∙K^−1^), but it has the drawback of low fracture toughness (typically 3–3.5 MPa·m^1/2^). When used in harsh working conditions of thermal shock and impact, such as those encountered in automobiles and wind power, AlN substrates are typically used in combination with plastic shock absorbers, which affects device miniaturization [6]. Due to their high theoretical thermal conductivity (320 W∙m^−1^∙K^−1^) and good fracture toughness (~10 MPa·m^1/2^) [7], Si_3_N_4_ ceramics have been regarded as the most promising alternatives for Al_2_O_3_ and AlN. However, the performances of Si_3_N_4_ ceramics currently available are far from theoretical optimization, which has limited their further applications.

The performance of Si_3_N_4_ ceramics is affected by various factors, among which their microstructure is a prominent factor [8,9]. The microstructure has been shown to directly influence the hardness and fracture toughness of Si_3_N_4_ [10,11,12,13,14]. At present, many researchers increase the fracture toughness of silicon nitride ceramics by adding reinforcements [15,16,17]. Guo et al. [18] prepared silicon nitride ceramics with high fracture toughness (9.7 MPa·m^1/2^) by taking Si_3_N_4_ as raw material and adding Lu_2_O_3_ as the reinforcing phase. However, adding the reinforcing phase cannot fundamentally solve the problem of low fracture toughness and high cost of silicon nitride ceramics.

Since the microstructure of Si_3_N_4_ ceramics is generally complex and uncontrolled, the effects of the phase composition of raw materials on the performances of Si_3_N_4_ have been thoroughly investigated [19,20]. Becher et al. [21] prepared Si_3_N_4_ ceramics with high fracture toughness (~10 MPa·m^1/2^) by sintering at 1850 °C under 1 MPa N_2_ atmosphere for 6 h, with α-Si_3_N_4_ and 2 vol% β-Si_3_N_4_ as raw materials. In order to ascertain the effects of β-Si_3_N_4_ seed crystal on the performances of Si_3_N_4_ ceramics, Peillon et al. [22] prepared Si_3_N_4_ ceramics with 2 vol% and 5 vol% of β-Si_3_N_4_ seed crystal and found that Si_3_N_4_ ceramics with 5 vol% of β-Si_3_N_4_ exhibited improved fracture toughness (8.4 MPa·m^1/2^). In their study, the fracture toughness increased by 30% as the sintering time increased by 3 h. Lee et al. [23] reported the synthesis of nanoscale α-Si_3_N_4_ with grain size distributions with two peaks when sintering was performed with the inclusion of β-Si_3_N_4_ (0%, 50%, 100%). The addition of β-Si_3_N_4_ led to improved performance of Si_3_N_4_ ceramics, where the sample with 50% of β-Si_3_N_4_ seed crystal exhibited the highest fracture toughness (7.9 MPa·m^1/2^). However, there is a need to further investigate the optimization of the β-Si_3_N_4_ seed crystal content since the high costs of nanoscale β-Si_3_N_4_ are not suitable for industrial applications.

Spark plasma sintering (SPS) or plasma-activated sintering, is a novel rapid hot-pressing sintering technique where particles are sintered using a pulse current, where plasma is generated by particle discharge under pulse current. Owing to its unique heating pattern, spark plasma can achieve rapid heating, which reduces the sintering time. Due to this feature, samples can be prepared with ultra-fine grain sizes [24,25]. Liu et al. [26] reported synthesis of Ybα-SiAlON with high density (3.42 g/cm^3^) and high fracture toughness (6.2 MPa·m^1/2^) by spark plasma sintering (1600 °C, 2 MPa, 5 min) with 5 wt% of Ybα-SiAlON as the seed crystal. Zamula et al. [27] prepared high-performance Si_3_N_4_ by spark plasma sintering (1800 °C, 20 min), wherein a complete transition from α-Si_3_N_4_ to β-Si_3_N_4_ was observed. The compactness of the prepared Si_3_N_4_ exceeded 98%, and its fracture toughness reached ~5.7 MPa·m^1/2^.

In this study, Si_3_N_4_ was prepared by SPS of α-Si_3_N_4_ (1650 °C, 30 MPa, 30 min) using a uniform-sized seed crystal of β-Si_3_N_4_ seed crystal. The microstructure of Si_3_N_4_ samples with different contents of β-Si_3_N_4_ seed crystal was investigated along with its correlation with hardness and fracture toughness. Insights obtained from this study can facilitate the design and preparation of Si_3_N_4_ with improved stability and effectiveness for applications in ceramic substrates.

## 2. Experimental

### 2.1. Materials

Mixtures of Si_3_N_4_ and sintering aid were used in this study. Two types of silicon nitride powders, 1# (α-Si_3_N_4_-rich powder) and 2# (β-Si_3_N_4_-rich powder) were used in the experiment. The powders had a nominal purity of over 90% and were purchased from Hebei Badu Metal Materials Co., Ltd. (Shijiazhuang, China). Nanoscale Y_2_O_3_ (purity > 99.99%), which served as the sintering aid, was purchased from Shanghai Yaoyi Alloy Materials Co., Ltd. (Shanghai, China). Al_2_O_3_ (0.2–0.4 μm, purity > 99.99%) was purchased from Hebei Yigui Welding Materials Co., Ltd. (Xingtai, China). PEG 400 (polyethylene glycol, purity > 99.99%), PAA (polyacrylic acid, purity > 99.99%), and ammonium citrate (purity > 99.99%) were purchased from Sinopharm Chemical Reagent Co., Ltd. (Shanghai, China).

### 2.2. Method

**Figure 1:**
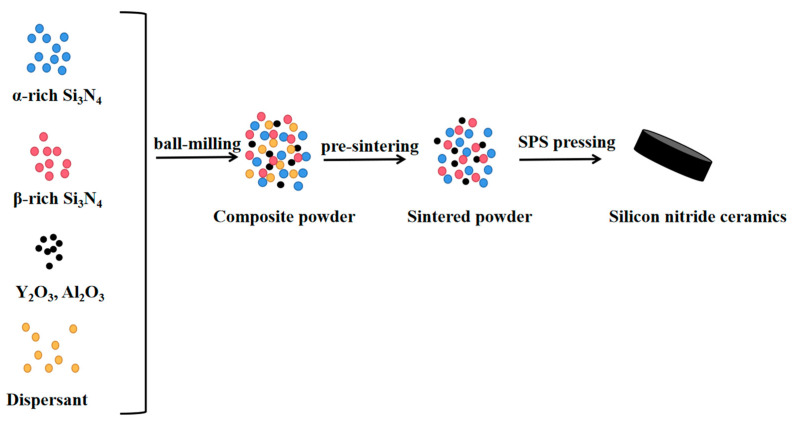
Schematic diagram of experimental process.

A mixture comprising 20 g of 1# and 2# Si_3_N_4_ was introduced into a beaker as indicated in Table 1. Subsequently, 360 μL of a solution containing PEG 400, ammonium citrate (dispersant), and 600 μL of PAA (adhesive) were added. To facilitate the sintering process, a total of 1.4 g of Y_2_O_3_ and 0.6 g of Al_2_O_3_ were introduced. The solution’s pH of 9.8 was modified and subsequently introduced into a polyurethane ball milling tank, containing zirconia grinding balls in a ball-to-powder weight ratio of 3:1. The wet-mixing process was carried out using pure water as the medium, and ball milling for 24 h. The compositions of various samples are provided in Table 1. Following ball milling, the slurry underwent vacuum drying at 120 °C for 24 h. After that, an 80-mesh sieve was utilized to obtain the Si_3_N_4_ composite precursor. The precursor was then subjected to pre-sintering at 800 °C for 2 h in a tube furnace, under an atmosphere of N_2_ (OTF- 1200X, Hefei Kejing Materials Co., Ltd., China). After pre-sintering, the product was again sieved through an 80-mesh sieve to acquire Si_3_N_4_ composite particles. Finally, the as-prepared Si_3_N_4_ composite was inserted into a graphite mold and subjected to Spark Plasma Sintering (SPS) at 1650 °C for 30 min while being subjected to a pressure of 30 MPa (SPS-5T-5-III, Shanghai Huachen Technology Co., Ltd., Shanghai, China). As shown in Figure 1.

### 2.3. Measurement

The sample density was measured by using the Archimedes method. The samples were analyzed using a Bruker D8 X-ray diffractometer equipped with an energy dispersive spectrometer (XRD, Karlsruhe, Germany) and a field-emission scanning electron microscope (SEM, FEI-Nova Nano 450, Hillsboro, OR, USA) to ascertain their phase compositions and fracture morphologies, respectively. The hardness of polished Si_3_N_4_ samples was tested using a Vickers hardness meter (HVS-50, Shanghai Wanheng Precision Instrument Co., Ltd., Shanghai, China) under a pressure of 1000 N for 15 s. Each sample was exposed to five replicates and the average value was taken as the final result. The fracture toughness of Si_3_N_4_ (*K_IC_*) was determined by using the Vickers hardness indentation method. The crack lengths of the samples were also determined. The fracture toughness can be calculated by:

(1) $$K_{IC}=0.075\times P\times c^{\frac{3}{2}}$$

where *K_IC_* is the fracture toughness (kgf·m^−3/2^), *P* is the load (kgf), and *c* is the diagonal length of the indentation crack (mm). 1 kgf·m^−3/2^ = 0.31 MPa·m^1/2^.

## 3. Results and Discussion

### 3.1. Phase Composition of Seed Crystal and Microstructure of Samples

**Figure 2:**
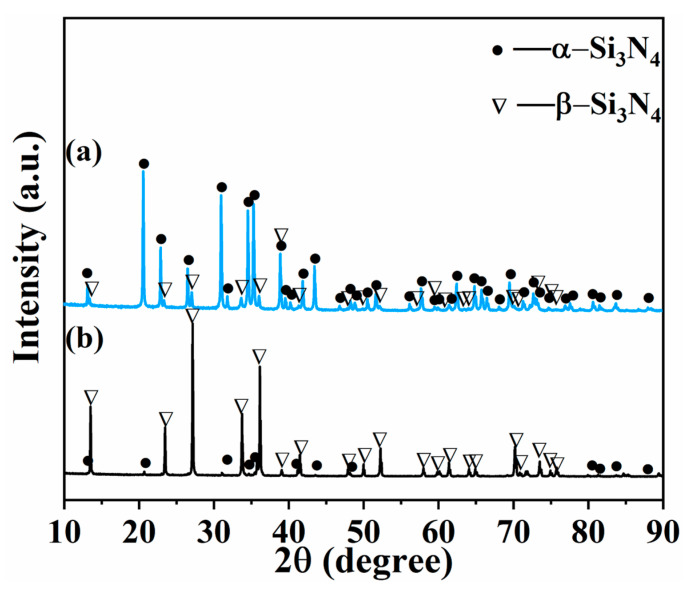
XRD patterns of raw powder: (**a**) 1#; (**b**) 2#.

XRD analyses were utilized for investigating the phase composition of the raw powders, as shown in Figure 2. The results indicate that both 1# and 2# samples were composed of hexagonal α-Si_3_N_4_ (JCPDS No.74-0554, a = b = 7.765 nm, c = 5.622 nm, α = β = 120°, γ = 90°) and hexagonal β-Si_3_N_4_ (JCPDS No.72-1308, a = b = 7.608 nm, c = 2.911 nm, α = β = 120°, γ = 90°). No other phases were observed. Among them, the main phase of 1# Si_3_N_4_ powder was α-Si_3_N_4_, while the main phase of 2# Si_3_N_4_ powder was β-Si_3_N_4_.

**Figure 3:**
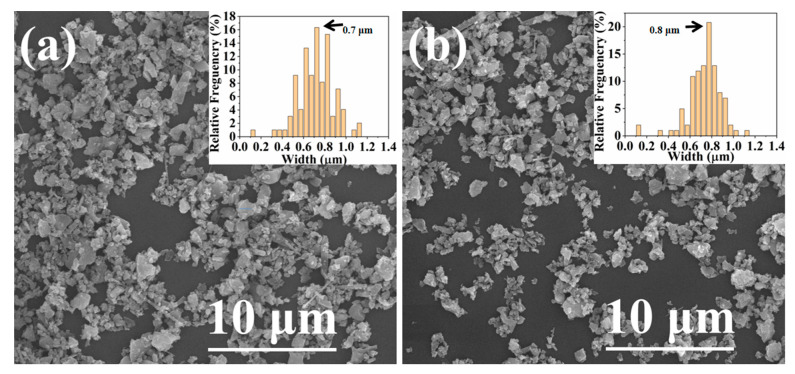
SEM images of Si_3_N_4_: (**a**) 1#; (**b**) 2#.

Figure 3 shows SEM images of raw powder samples of α-rich Si_3_N_4_ and β-rich Si_3_N_4_. Raw powder samples of α-rich Si_3_N_4_ comprised irregularly shaped particles that were observed to aggregate (size = 0.5–1 μm), as shown in Figure 3a. Combined with the analysis of XRD patterns (Figure 2), it can be concluded that the observed rod-like powder was β-Si_3_N_4_. Raw powder samples of β-rich Si_3_N_4_ also comprised irregularly shaped particles with aggregation (size = 0.5–1 μm), as shown in Figure 3b. Overall, the two powders had consistent shapes and sizes, and the particles were found to be well dispersed with negligible agglomeration.

### 3.2. Effects of Seed Crystal Composition on Phase Composition and Microstructure of Ceramic Samples

**Figure 4:**
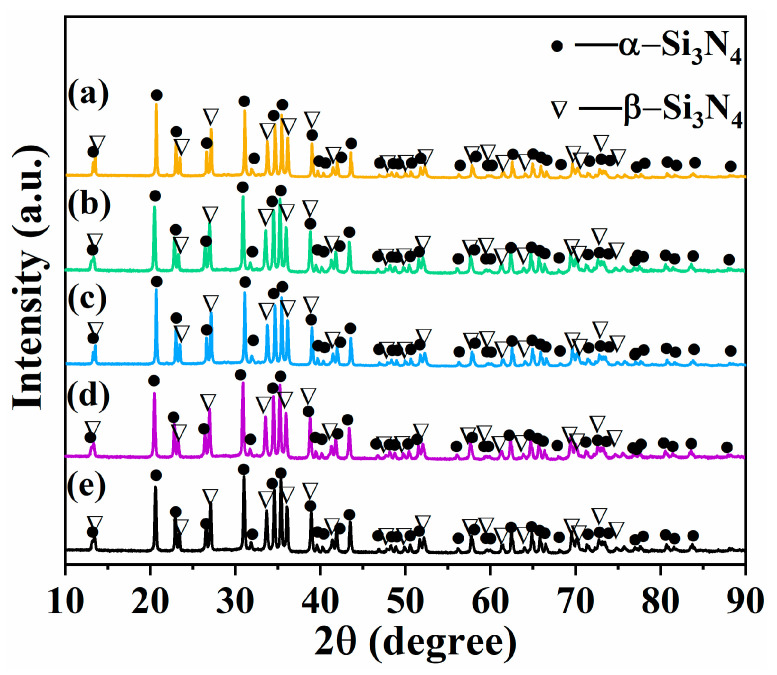
XRD patterns of Si3N4 samples prepared by SPS at 1100 °C: (**a**) SN0; (**b**) SN1; (**c**) SN2; (**d**) SN3; (**e**) SN4.

XRD patterns of samples with different contents of β-Si_3_N_4_ seed crystal (SN0~SN4) prepared by SPS at 1100 °C exhibited characteristic peaks corresponding to hexagonal α-Si_3_N_4_ (JCPDS No.74-0554, a = b = 7.765 nm, c = 5.622 nm, α = β = 120°, γ = 90°) and hexagonal β-Si_3_N_4_ (JCPDS No.72-1308, a = b = 7.608 nm, c = 2.911 nm, α = β = 120°, γ = 90°), as shown in Figure 4.

**Figure 5:**
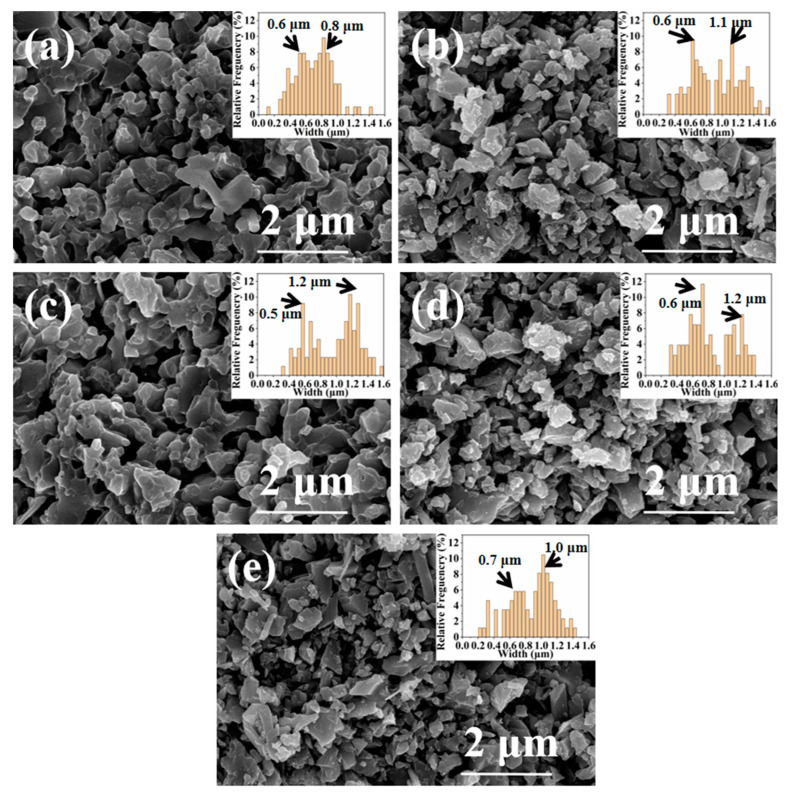
SEM images of sample fractures prepared by SPS at 1100 °C: (**a**) SN0; (**b**) SN1; (**c**) SN2; (**d**) SN3; (**e**) SN4. Insets show the size distributions of grains.

Figure 5 illustrates the grain size changes in SN0~SN4 ceramic samples prepared by SPS at 1100 °C. In the cases with few amounts of β-Si_3_N_4_ seeds, e.g., sample SN0 (Figure 5a), the grain size is relatively small; in the cases with a few amounts of β-Si_3_N_4_, e.g., sample SN1~SN3 (Figure 5b–d), some grains become smaller while the other grains grew, the grain size variation in SN4 samples is small.

**Figure 6:**
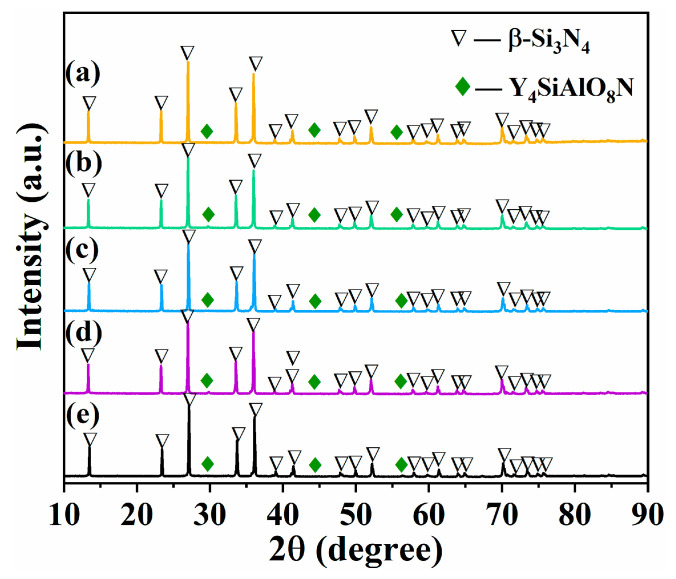
XRD patterns of Si_3_N_4_ samples prepared by SPS at 1650 °C: (**a**) SN0; (**b**) SN1; (**c**) SN2; (**d**) SN3; (**e**) SN4.

XRD patterns of samples with different contents of β-Si_3_N_4_ seed crystal (SN0~SN4) prepared by SPS at 1650 °C exhibited characteristic peaks corresponding to hexagonal β-Si_3_N_4_ (JCPDS No.72-1308) and hexagonal Y_4_SiAlO_8_N (JCPDS No.48-1630), as shown in Figure 6. No characteristic peaks of Al_2_O_3_ and Y_2_O_3_ sintering aids were detected. Complete phase transition of α-Si_3_N_4_ into β-Si_3_N_4_ and transition of Al_2_O_3_ and Y_2_O_3_ into Y_4_SiAlO_8_N solid solution were observed in the composite powder. Since the bond lengths of Si-N and Al-O were 0.174 nm and 0.175 nm, respectively, it is speculated that the Al-O bond may replace the Si-N bond during the sintering process. This resulted in the generation of Y_4_SiAlO_8_N solid solution via reaction with Y_2_O_3_, suggesting that Y, Al, and O were all present in the lattice structure of β-Si_3_N_4_ giving rise to an interfacial phase. This speculation is consistent with the observations from earlier reports [28,29].

**Figure 7:**
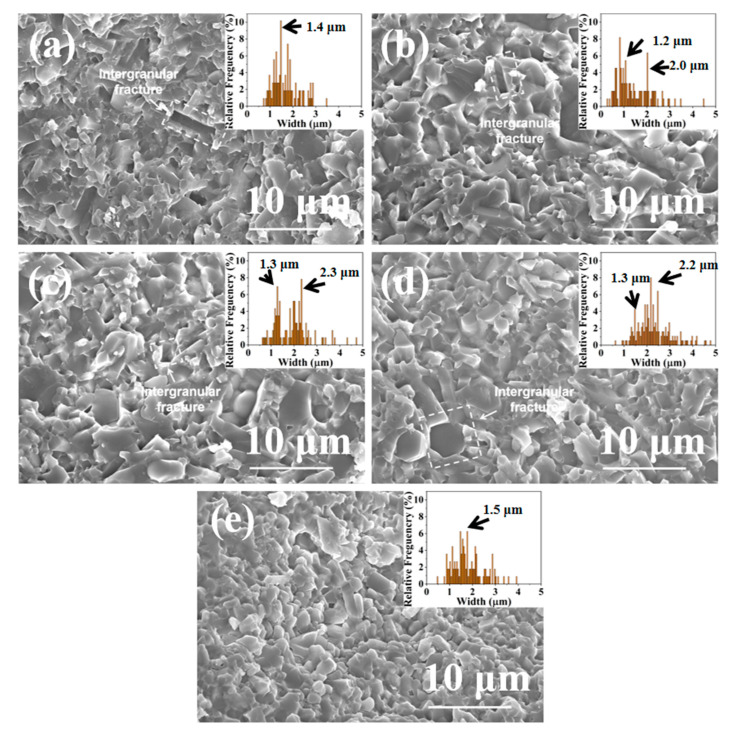
SEM images of sample fractures prepared by SPS at 1650 °C: (**a**) SN0; (**b**) SN1; (**c**) SN2; (**d**) SN3; (**e**) SN4. Insets show the size distributions of grains.

The grain size distribution of Si_3_N_4_ significantly impacts its fracture toughness. Figure 7 illustrates the fracture morphologies of SN0-SN4 samples prepared by SPS at 1650 °C. As can be observed in Figure 7a, the SN0 sample comprised matrix grains (with ~1.4 μm granular grains), large grains (rod-like grains with a length of about 2 μm, width of about 1 μm), and abundant pores (~2 μm), a uniform distribution of large and small grains was observed. The SN1 sample comprised matrix grains (~1.3 μm granular grains), large grains (rod-like grains with a length of about 2 μm, width of about 1 μm), and a small number of pores (~1 μm), as shown in Figure 7b, the size distribution exhibited two peaks, where again the large and small grains were uniformly distributed. The SN2 sample comprised matrix grains (with ~1.3 μm granular grains and a small amount of rod-like grains with a length of about 2.2 μm, width of about 1 μm), large grains (rod-like grains with a length of about 6 μm, width of about 2.3 μm), and a small number of pores (~1 μm), as shown in Figure 7c, the size distribution exhibited two peaks, herein, the large and small grains were uniformly distributed in an interlocked structure. The SN3 sample comprised matrix grains (with ~1.3 μm granular grains and a small amount of rod-like grains with a length of about 1 μm, width of about 0.5 μm), large grains (rod-like grains (length of about 8 μm, width of about 2.2 μm)), and a small number of pores (~1 μm), as shown in Figure 7d, similar to the case of SN2, large and small grains were uniformly distributed in an interlocked structure. The SN4 sample comprised matrix grains (with granular grains (~1 μm) and rod-like grains with a length of about 1 μm, width of about 0.5 μm), large grains (with granular grains (~1.5 μm) and rod-like grains with a length of about 1.5 μm, width of about 0.5 μm), and a small number of pores (~1 μm), as shown in Figure 5e, similar to the other samples, the large and small grains were uniformly distributed. Additionally, sample failure was dominated by intergranular fracture.

With the increase in β-Si_3_N_4_ seed content, the grain distribution gradually changed from a single peak distribution (Figure 7a) to a bimodal distribution (Figure 7c). When excess β-Si_3_N_4_ seeds were introduced, the grain distribution changed from a bimodal distribution (Figure 7c) back to a unimodal distribution (Figure 7e).

The elemental composition of the SN0–SN4 samples was verified through the utilization of an EDX analysis, as shown in Table 2. The Table demonstrates a uniform distribution of Si, N, Al, Y, and O elements within the silicon nitride matrix. The proportion of each element in SN0~SN4 ceramic samples is similar.

### 3.3. Effect of β-Si_3_N_4_ Seed on the Sintering and Grain Growth of Silicon Nitride Ceramics

During the liquid phase sintering process, α-Si_3_N_4_ in the original powder gradually dissolves into the liquid phase aid and diffuses. Its reprecipitation mainly occurs in two ways. The first is through uniform nucleation and growth, where small-sized β-Si_3_N_4_ particles are formed. The second involves non-uniform nucleation and growth in the high-energy crystal plane (rod-shaped axis) of β-Si_3_N_4_ seeds, which causes the β-Si_3_N_4_ seeds to grow into rod-shaped crystals.

**Figure 8:**
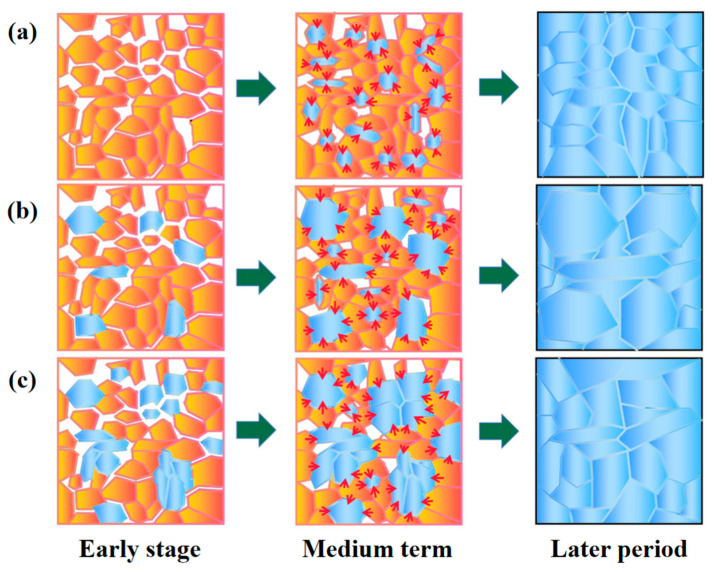
Representative figure of sintering mechanism of silicon nitride ceramics: (**a**) 0% of β-Si_3_N_4_ seed crystal; (**b**) 20% of β-Si_3_N_4_ seed crystal; (**c**) 50% of β-Si_3_N_4_ seed crystal. (“early stage” refers to the original powder before sintering; “medium term” refers to the microstructure change of silicon nitride ceramics sintered at 1100 °C; “later period” refers to the microstructure change of silicon nitride ceramics sintered at 1650 °C).

The influence of crystal seed phase composition on the microstructure of ceramics is shown in Figure 7. As shown in Figure 8a, when 0% β-Si_3_N_4_ crystal seeds are introduced, α-Si_3_N_4_ mainly transforms into β-Si_3_N_4_ following a uniform nucleation growth mode, which results in the formation of uniform grains with fine grain size (Figure 7a).

As shown in Figure 8b, when an appropriate amount (20%) of β-Si_3_N_4_ seeds is introduced, during the dissolution precipitation process, α-Si_3_N_4_ exhibits both uniform nucleation and growth as well as non-uniform nucleation. According to the Ostwald ripening theory [30,31], β-Si_3_N_4_ particles in close contact with α-Si_3_N_4_ precipitate on the surface of the original β-Si_3_N_4_ seeds, leading to the formation of large grains. At the same time, a portion of α-Si_3_N_4_ precipitates and grows uniformly, forming an interlocking structure with the generated large extension grains (Figure 7c).

As shown in Figure 8c, when an excess amount (50%) of β-Si_3_N_4_ crystal seeds is introduced, non-uniform nucleation of β-Si_3_N_4_ occurs during the dissolution precipitation process. Due to the introduction of excessive β-Si_3_N_4_ crystal seeds into the original powder, the β-Si_3_N_4_ crystal seeds come into contact with each other, resulting in a decrease in α-Si_3_N_4_ on the surface of the β-Si_3_N_4_ crystal seeds. The large particles formed by precipitation on the surface of the β-Si_3_N_4_ crystal seeds are reduced, which is similar in size to the β-Si_3_N_4_ particles formed by uniform nucleation and growth, resulting in a more uniform size of grain growth (Figure 7e).

### 3.4. Effects of Seed Crystal Composition on Sample Performances

**Figure 9:**
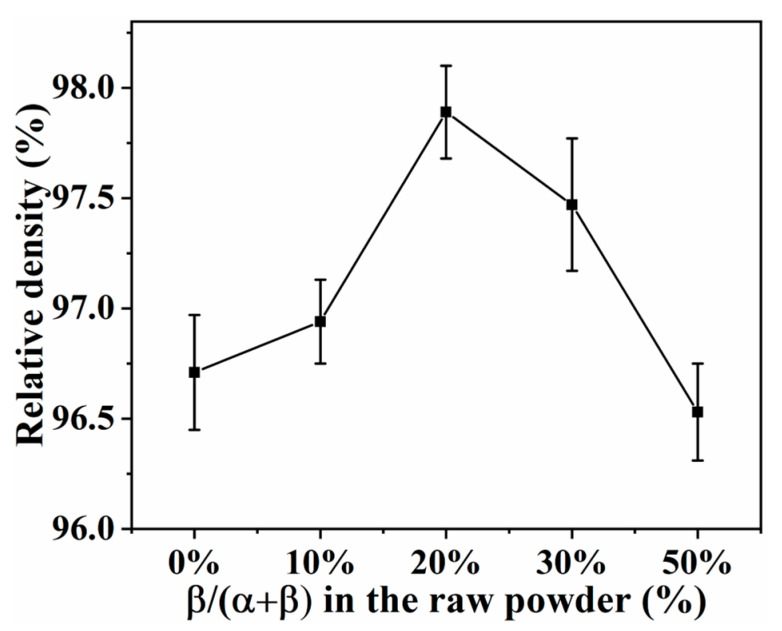
Effects of content of β-Si_3_N_4_ seed crystal on relative density of Si_3_N_4_ prepared by SPS at 1650 °C.

Figure 9 shows the trends of relative density of SN0~SN4 prepared by SPS at 1650 °C. As the content of β-Si_3_N_4_ seed crystal increased from 0% to 20%, the relative density of Si_3_N_4_ samples increased from 96.7% to 97.9%. This was suggestive of the fact that grain size distribution with two peaks favors high compactness of ceramics (see fracture morphology, Figure 7). As the content of β-Si_3_N_4_ seed crystal increased from 20% to 50%, the relative density of Si_3_N_4_ samples decreased from 97.9% to 96.5%, which may be attributed to the degrading grain size distribution with two peaks (see fracture morphology, Figure 7).

**Figure 10:**
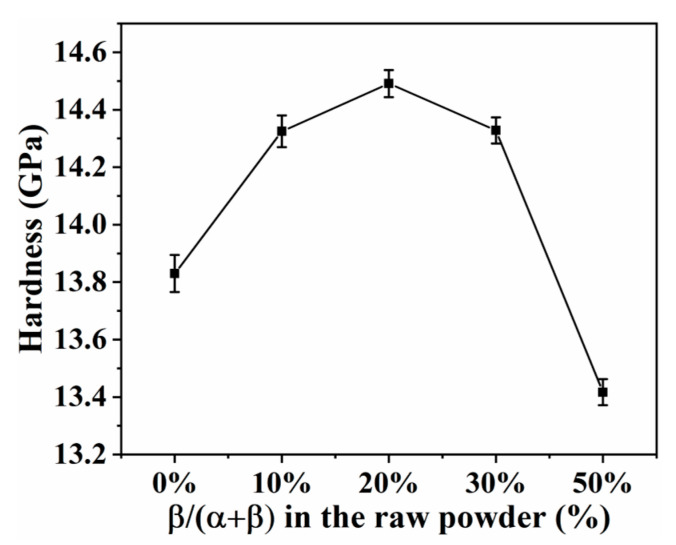
Effects of content of β-Si3N4 seed crystal on Vickers hardness of Si_3_N_4_ prepared by SPS at 1650 °C.

Figure 10 shows the Vickers hardness of SN0–SN4 samples prepared by SPS at 1650 °C. As the content of β-Si_3_N_4_ seed crystal was increased from 0% to 20%, the Vickers hardness of Si_3_N_4_ samples increased from 13.8 GPa to 14.5 GPa, emphasizing the significance of two peaks in the grain size distribution (see fracture morphology, Figure 7). When the content of β-Si_3_N_4_ seed crystal was increased from 20% to 50%, the Vickers hardness of Si_3_N_4_ samples decreased from 14.5 GPa to 13.4 GPa, which was attributed to degradation in the grain size distribution with two peaks and grain interlocking structure (see fracture morphology, Figure 7). Hence, it can be concluded that the two peaks observed in the grain size distribution have a major influence on the mechanical properties of the samples (see fracture morphology, Figure 4). Among all the samples tested, the SN2 sample exhibited the highest Vickers hardness (14.5 GPa).

**Figure 11:**
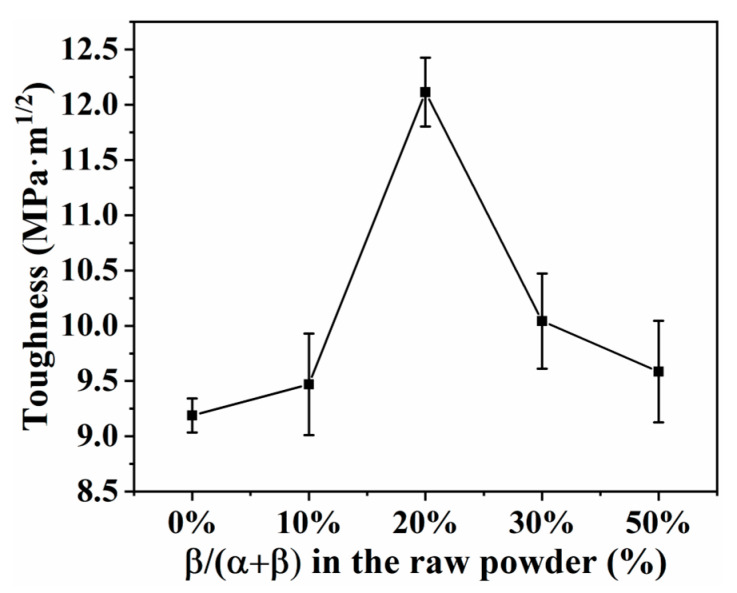
Effects of content of β-Si3N4 seed crystal on fracture toughness of Si_3_N_4_ prepared by SPS at 1650 °C.

Figure 11 shows the fracture toughness of SN0–SN4 samples prepared by SPS at 1650 °C. As the content of β-Si_3_N_4_ seed crystal was increased from 0% to 20%, the fracture toughness of Si_3_N_4_ samples increased from 9.2 MPa·m^1/2^ to 12.1 MPa·m^1/2^. This was ascribed to the emergence of intergranular fracture (see fracture morphology, Figure 7). As the content of β-Si_3_N_4_ seed crystal was increased from 20% to 50%, the fracture toughness of Si_3_N_4_ samples decreased from 12.1 MPa·m^1/2^ to 9.5 MPa·m^1/2^ (see fracture morphology, Figure 7). The decrease in Vickers hardness may be related to the degradation in the grain size distribution with two peaks and grain interlocking structure. Among all samples examined, the SN2 sample exhibited the highest fracture toughness (12.1 MPa·m^1/2^). This was attributed to its unique grain size distribution with two peaks and grain interlocking structure.

In the SN0 sample, due to the presence of small and equiaxed β-Si_3_N_4_ grains, the crack deflection during fracturing was low, which resulted in low fracture toughness. In the SN1 sample, the β-Si_3_N_4_ grains consisted of small and equiaxed rod-like grains, which also led to low fracture toughness. However, for the SN2 sample, the grain size distribution exhibited two peaks, where the large and small grains formed an interlocking structure. This feature resulted in low porosity and imparted the sample with high relative density (97.9%) and the highest Vickers hardness (14.5 GPa) among all the samples, as shown in Figure 7. Herein, the heterogeneous precipitation of α-Si_3_N_4_ on the surface of β-Si_3_N_4_ seed crystals led to the formation of large, rod-like grains, which activated grain bridging and extrusion, and ultimately resulted in the self-toughening of Si_3_N_4_ samples. The fracture toughness of the SN2 sample reached 12.1 MPa·m^1/2^, as shown in Figure 10. With excess β-Si_3_N_4_ seed crystals (30–50%), the original β-Si_3_N_4_ seed crystals had consistent sizes with β-Si_3_N_4_ particles generated by homogeneous phase transition of α-Si_3_N_4_, resulting in low crack deflection during fracturing, which drastically reduced the fracture toughness.

## 4. Conclusions

Si_3_N_4_ ceramics with different contents of β-Si_3_N_4_ seed crystal were prepared using the spark plasma sintering method, and the correlation of the β-Si_3_N_4_ content with hardness and fracture toughness was investigated. The grain growth in Si_3_N_4_ was found to be controlled by the dissolution-precipitation process. All the as-prepared samples comprised small particles (diameter about 1 μm) and large particles (about 2 μm). Additionally, the large and small grains were observed to be uniformly distributed. Results showed that a suitable content of (≤20%) β-Si_3_N_4_ seed crystal could induce the phase transition from α-Si_3_N_4_ to rod-like β-Si_3_N_4_, which resulted in the grain size distribution exhibiting two peaks. At this optimized β-Si_3_N_4_ seed crystal content of 20%, the best overall mechanical performance was achieved. At higher content of β-Si_3_N_4_ seed crystal (>50%), the β-Si_3_N_4_ particles showed a tendency to be mutually constrained during sintering, with uniform size distribution and lower performance. Among samples prepared by the spark plasma sintering method (1650 °C, 30 MPa, 30 min), the sample with a β-Si_3_N_4_ seed crystal content of 20% exhibited the best overall performance with a density of 97.9%, hardness of 14.5 GPa, and fracture toughness of 12.1 MPa·m^1/2^.

The optimized Si_3_N_4_ in this work (using 20% β-Si_3_N_4_ as seed crystal) presents the highest toughness but the lowest hardness among previous works [32,33,34,35] as shown in Table 3. This work may provide a potential application of ceramics substrate in high-speed rail and new energy vehicles. Further verification of the actual use of silicon nitride ceramics by researchers in the field of automotive development is needed.

## Figures and Tables

**Table 1:** Compositions of different Si_3_N_4_ samples.

Sample	Fraction of 1#	Fraction of 2#
SN0	100%	0%
SN1	90%	10%
SN2	80%	20%
SN3	70%	30%
SN4	50%	50%

**Table 2:** SEM-EDX relative content of various elements in silicon nitride ceramics prepared by SPS at 1650 °C.

Sample	Si (%)	N (%)	O (%)	Y (%)	Al (%)
SN0	46.3	43.7	6.6	1.9	1.5
SN1	47.2	43.8	6.2	1.7	1.1
SN2	46.1	43.8	6.7	1.8	1.6
SN3	46.7	43.5	6.4	2	1.4
SN4	46.9	43.2	6.6	1.7	1.6

**Table 3:** Comparison of fracture toughness with other silicon nitride ceramics.

Raw Material	Preparation Method	Relative Density (%)	Hardness (GPa)	Toughness (MPa·m^1/2^)	Reference
80% α-Si_3_N_4_ and 20% β-Si_3_N_4_	SPS, 1650 °C, 30 MPa, 30 min	97.9	14.5	12.1	this paper
α-Si_3_N_4_	HP, 1620 °C, 30 MPa, 3 h	92.1	12.5	6.02	[32]
90% α-Si_3_N_4_ and 10% SiC ceramic composites	HP, 1680 °C, 34 MPa, 4 h	97.9	16.4	8.2	[33]
70% α-Si_3_N_4_ and 30% ZrO_2_ ceramic composites	SPS, 1600 °C, 30 MPa, 10 min	99	13.2	7.1	[34]
90% α-Si_3_N_4_ and 10% Ti(C, N) ceramic composites	SPS, 1700 °C, 50 MPa, 6 min	99.7	15.6	8.3	[35]

Note: SPS (spark plasma sintering); HP (Hot pressing sintering).

## Data Availability

All data generated or analyzed during this study are included in this published article.
